# Supplementary material for: Case-control study of patient characteristics, knowledge of the COVID-19 disease, risk behaviour and mental state in patients visiting an emergency room with COVID-19 symptoms in the Netherlands
Source: PLoS One. 2021 Apr 28;16(4):e0249847. doi: 10.1371/journal.pone.0249847 (PMC8081234; doi:10.1371/journal.pone.0249847)
Supplement: S1 Questionnaire — (DOCX) [file pone.0249847.s001.docx]

Attachment 1.

**Questionnaire on knowledge and illness perception**

**Knowledge:**

1. Most people become infected with SARS-Cov-2 virus through:

- Animals
- Other people
- Contaminated material

1. SARS-Cov-2 virus is comparable in severity with Influenza:

- Less severe
- Comparable
- More severe

1. Only old people can be infected with SARS-Cov-2 virus:

- Not true
- True

1. Children can transmit the SARS-Cov-2 infection:

- Not true
- True

1. The greatest risk to dye by SARS-Cov-2 virus have:

- Children
- Adults
- Seniors

1. Stick to government rules (for example: keep 1.5-meter distance) helps in preventing infection:

- Not true
- True

1. It is not possible to be infected by SARS-Cov-2 virus if you wear a face mask:

- Not true
- True

**Illness perception**

1. I thought not to become infected with the SARS-Cov-2 virus:

- Not true
- True

1. If you are infected with SARS-Cov-2 virus, do you think that you will become seriously ill?

- Not true
- True

1. If you are infected with SARS-Cov-2 virus, do you think that you can die by this infection?

- Not true
- True

1. If you are infected with SARS-Cov-2 virus, do you think you are contagious?

- Not true
- True
