# Supplementary material for: Case-control study of patient characteristics, knowledge of the COVID-19 disease, risk behaviour and mental state in patients visiting an emergency room with COVID-19 symptoms in the Netherlands
Source: PLoS One. 2021 Apr 28;16(4):e0249847. doi: 10.1371/journal.pone.0249847 (PMC8081234; doi:10.1371/journal.pone.0249847)
Supplement: S2 Questionnaire — (DOCX) [file pone.0249847.s002.docx]

Attachment 2.

**Questionnaire on risk behaviour**

**(during the COVID-19 crisis: 1th of March to 1th of July 2020)**

**Hygiene**

1. **How many times do you wash your hands?**
2. Never 0 1-3 x/day 0 4-6 x/day 0 7-9 x/day 0 >10 x/day
3. **Do you wash your hand more than 20 seconds?**

0 Never 0 Almost never 0 Sometimes 0 Almost always 0 Always

1. **Do you sneeze and cough in the inside of your elbow?**

0 No 0 Probably not 0 Possible 0 Probably yes 0 Yes

1. **Do you wear a face mask if you go outside?**

` 0 Never 0 Almost never 0 Sometimes 0 Almost always 0 Always

**Human contact**

1. **Do you shake hands?**

0 No 0 Yes

1. **Do you hold on 1.5-meter distance between you and other persons?**

0 Never 0 Almost never 0 Sometimes 0 Almost always 0 Always

1. **Do you stay at home with your symptoms?**

0 Never 0 Almost never 0 Sometimes 0 Almost always 0 Always

1. **Did you go to sport activities outside before you had symptoms ?**

0 Never 0 Almost never 0 Sometimes 0 Almost always 0 Always

1. **Did you stay at home before you had your symptoms?**

0 Never 0 Almost never 0 Sometimes 0 Almost always 0 Always

1. **If a family member had symptoms, do you stay at home?**

0 No 0 Probably not 0 Possible 0 Probably yes 0 Yes

1. **If a family member had symptoms, do you hold 1.5-meter distance?**

0 No 0 Probably not 0 Possible 0 Probably yes 0 Yes

**Social distancing**

1. **Do you visit family?**

0 No 0 Yes

1. **Do you visit shops (beside the basic necessities of life)?**

0 No 0 Yes

1. **Do you visit markets, parks or beaches?**

0 No 0 Yes

1. **Do you visit people > 65 years old?**

0 No 0 Yes

1. **Do you receive people > 65 years old?**

0 No 0 Yes

1. **Do you visit groups of people?**

0 No 0 max 2 0 3-5 0 6-10 0 >10
