# Supplementary material for: Case-control study of patient characteristics, knowledge of the COVID-19 disease, risk behaviour and mental state in patients visiting an emergency room with COVID-19 symptoms in the Netherlands
Source: PLoS One. 2021 Apr 28;16(4):e0249847. doi: 10.1371/journal.pone.0249847 (PMC8081234; doi:10.1371/journal.pone.0249847)
Supplement: S1 Protocol — (PDF) [file pone.0249847.s003.pdf]

Questionnaire not subject to WMO research

Enclose this completed questionnaire with the other documents of your non-WMO application. For more information, see: https://www.mec-u.nl/wmo/niet-wmo-impig-onderzoek/

Date of completing form 08-04-20

Completed by JPM-Kuiper van der Valk, MD, PHD Pulmonary Diseases Franciscus Gasthuis & Vlietland

The title of the research is:

***P**atient's knowledge **A**nd behavior o**N** the COVID-19 disease and as **DE**ter**MI**nants of **C**ontamination (PANDEMIC-study)*

Give a short Dutch summary of the research (describe what will happen in the research, including method; minimum 8 lines / max. 250 words):

Achtergrond: Het Coronavirus 2019 (COVID-19) is de meest ernstigste respiratoire pandemie sinds de H1N1-grieppandemie van 1918. Bij afwezigheid van een COVID-19-vaccin zijn niet-farmaceutische interventies (NFI's) cruciaal om de overdracht van virussen te verminderen. Het belang van het naleven van de door de overheid opgelegde interventies wordt van cruciaal belang geacht. Kennis over besmettingsrisico's en over de ernst, de mogelijke gevolgen, de kans op herstel en de langetermijngevolgen van de ziekte zal per persoon verschillen. Risicogedrag zal van invloed zijn op het volgen van interventies zoals het isoleren van personen, quarantaine thuis, sociale afstand houden (vooral van mensen ouder dan 70 jaar) en het vermijden van massa bijeenkomsten.
We veronderstellen dat de kennis van de patiënt over de COVID-19-virusinfectie en (risico) gedrag verband houdt met het infectierisico voor het COVID-19-virus.

Doel: Het primaire doel is om de kennis van de patiënt over de ziekte en hun risicogedrag te bestuderen. Het secundaire doel is om het niveau van deze kennis en risicogedrag (vasthouden aan NPI's) te relateren aan besmetting.

Methode: We zullen ten minste 170 patiënten (2 groepen, verschil in ziekteperceptie van 20%, 90% power en alfa 0,05) includeren. De patiënten worden onderworpen aan een COVID-19-infectie screening op de eerstehulpafdeling of de COVID-19-afdeling. Na het geven van geïnformeerde toestemming, zullen patiënten worden gevraagd om een drietal vragenlijsten in te vullen (op een iPad met wegwerphoes) over kennis van de ziekte, over hun risicogedrag en gemoedstoestand. De relatie tussen ziekte-inzicht en risicogedrag zal worden onderzocht in de COVID-19 positieve groep en zal worden vergeleken met zowel de COVID-19 negatieve groep als met historische gegevens uit de literatuur.

Het onderzoek is niet belastend voor de patiënt en op korte termijn haalbaar.
De gegevens worden anoniem verwerkt en er wordt een patiënten lijst bijgehouden door 1 arts en bewaard in een Excel bestand op een vergrendelde schijf.

Resultaten: De resultaten van de vragenlijsten worden beschrijvend gepresenteerd en worden gecorreleerd aan besmettingsrisico's. De resultaten van de studie zullen het belang van het volgen van opgelegde interventies vermoedelijk onderbouwen. De uitkomst van dit

onderzoek zal steun kunnen geven aan overheidscampagnes bij het verstrekken van informatie aan burgers.

The expected start date is [13-04-2020]

The expected completion date is [13-08-2020]

Please answer the following questions:

1. Is a medical device used during the examination?
☐ Yes ☒ No ☐ N / A

If yes, please complete the following information:

This medical device may / will not be CE marked and will / will not be used within the intended use by the manufacturer.

Not applicable

2. Why do you think the research does not fall under the Medical Scientific Research Act (WMO) involving people (please tick as appropriate)?

☐ Further use is made of (medical) data of people who have already been or will be recorded for another purpose.

☐ Further use is made of human tissue that has already been or is being collected for a purpose other than scientific research or that remains after diagnosis or treatment.

☒ Other, namely: Non-invasive survey questionnaire study

3. Is it a study in which patients / test subjects are included?

☒ Yes ☐ No.

If yes, indicate how many participants are involved and type the population:

The population concerns: 170 patients with suspected COVID-19 (almost all presentations for the lung diseases at the emergency room, because if someone coughs is considered suspicious) are included and data are used from these patients. We expect a distribution of 40% negative and 60% positive Covid-19 patients.

1. Is the research in which test subjects are subjected to (an) action (s) or are imposed on them a code of conduct (including the administration of questionnaire (s))?

☒ Yes ☐ No.

If so, which action (s) or rules of conduct do not occur in the context of the standard treatment of the test subject?

Questionnaires

If yes, please add the questionnaires and / or interview questions to your submission!

1. What is the (expected) burden for the participants:

☒ Taxation in time / duration, namely ___ 15 minutes
☒ Psychological burden, namely: ___the patient has to concentrate on the A&E during his presentation to answer the questions.
☐ Physical load, namely ___No____

2. How are the participants approached and informed?

Patients are informed by a physician assistant about the burden, number of questionnaires and the content of the questions. This takes place during the waiting period until the additional examination of the regular care is known.

No information letter and consent form apply to this non-WMO application.

A waiver will be used because of several investigations that are ongoing at the emergency unit regarding the corona virus.
We will, however, ask the patients permission in the first question in the questionnaire to participate in the study.

Please note: also in case of file research, information must be provided about the research and written permission must be requested, unless this is impossible in practice.

If not, the reason for this is that:
☐ the number of participants to whom consent must be sought is so large that it is impossible to do so.
Please note: you have a best efforts obligation under the GDPR!

☐ the data / human tissue can no longer be traced back to a person in any way.

☐ other reason, namely _____________

Not applicable

Data principal investigator

Sir / madam, titles and name: Kuiper-van der Valk, MD, PhD

Position / department / institution: AIOS + Post Doc Lung Diseases

E-mail address: h.kuiper-vandervalk@franciscus.nl

Postal address (including room number): Kleiweg 500, 3045 PM, Rotterdam. Room 00D41

Signature:

Who is the initiator of the research?

(*Franciscus Gasthuis en Vlietland (ziekenhuis)*

Invoice details

For applications from a client who is not affiliated with MEC-U (both other healthcare institutions and industry) costs will be charged.

The billing address is:

Franciscus Gasthuis & Vlietland

JPM Kuiper van der Valk

Cost number: 800521

Kleiweg 500 3045 PM Rotterdam

If the invoice details are not complete, we will not process your request!

Is it a doctoral research or part of a doctoral research?

☐ Yes ☒ No.

If so, name of doctoral candidate / institution / department:

Does the research concern a graduation project or an internship research?

☐ Yes ☒ No.

If so, name of student / intern / training institute / study program:

Any other person (s) involved
Name of Institution (s): Department (s)

A.W.M. Evers, Prof, PhD **
M. Middendorp, Assoc. Prof, PhD **
Dr. J.C.C.M. in 't Veen, MD, PhD *

*Department of Pulmonary Medicine Franciscus Gasthuis & Vlietland
** Department of Medical and Neuropsychology University Leiden
