# Supplementary material for: Case-control study of patient characteristics, knowledge of the COVID-19 disease, risk behaviour and mental state in patients visiting an emergency room with COVID-19 symptoms in the Netherlands
Source: PLoS One. 2021 Apr 28;16(4):e0249847. doi: 10.1371/journal.pone.0249847 (PMC8081234; doi:10.1371/journal.pone.0249847)
Supplement: S2 Protocol — (PDF) [file pone.0249847.s004.pdf]

Vragenlijst niet WMO-plichtig onderzoek

Deze ingevulde vragenlijst meesturen met de overige documenten van uw niet-WMO-aanvraag. Zie voor meer informatie: https://www.mec-u.nl/wmo/niet-wmo-plichtig-onderzoek/

Datum invullen formulier 08-04-20]

Ingevuld door JPM-Kuiper van der Valk, MD, PH Longziekten Franciscus Gasthuis& Vlietland

De **titel** van het onderzoek is

***P**atient's knowledge **A**nd behavior o**N** the COVID-19 disease and as **DE**ter**MI**nants of **C**ontamination (PANDEMIC-study)*

Geef een korte Nederlandse **samenvatting** van het onderzoek (beschrijf wat er gaat gebeuren in het onderzoek, inclusief methode; minimaal 8 regels/max. 250 woorden):

Achtergrond: Het Coronavirus 2019 (COVID-19) is de meest ernstigste respiratoire pandemie sinds de H1N1-grieppandemie van 1918. Bij afwezigheid van een COVID-19-vaccin zijn niet-farmaceutische interventies (NFI's) cruciaal om de overdracht van virussen te verminderen. Het belang van het naleven van de door de overheid opgelegde interventies wordt van cruciaal belang geacht. Kennis over besmettingsrisico's en over de ernst, de mogelijke gevolgen, de kans op herstel en de langetermijngevolgen van de ziekte zal per persoon verschillen. Risicogedrag zal van invloed zijn op het volgen van interventies zoals het isoleren van personen, quarantaine thuis, sociale afstand houden (vooral van mensen ouder dan 70 jaar) en het vermijden van massa bijeenkomsten.
We veronderstellen dat de kennis van de patiënt over de COVID-19-virusinfectie en (risico) gedrag verband houdt met het infectierisico voor het COVID-19-virus.

Doel: Het primaire doel is om de kennis van de patiënt over de ziekte en hun risicogedrag te bestuderen. Het secundaire doel is om het niveau van deze kennis en risicogedrag (vasthouden aan NFI's) te relateren aan besmetting.

Methode: We zullen ten minste 170 patiënten (2 groepen, verschil in ziekteperceptie van 20%, 90% power en alfa 0,05) includeren. De patiënten worden onderworpen aan een COVID-19-infectie screening op de eerstehulpafdeling of de COVID-19-afdeling. Na het geven van geïnformeerde toestemming, zullen patiënten worden gevraagd om een drietal vragenlijsten in te vullen (op een iPad met wegwerphoes) over kennis van de ziekte, over hun risicogedrag en gemoedstoestand. De relatie tussen ziekte-inzicht en risicogedrag zal worden onderzocht in de COVID-19 positieve groep en zal worden vergeleken met zowel de COVID-19 negatieve groep als met historische gegevens uit de literatuur.

Het onderzoek is niet belastend voor de patiënt en op korte termijn haalbaar.
De gegevens worden anoniem verwerkt en er wordt een patiënten lijst bijgehouden door 1 arts en bewaard in een Excel bestand op een vergrendelde schijf.

Resultaten: De resultaten van de vragenlijsten worden beschrijvend gepresenteerd en worden gecorreleerd aan besmettingsrisico's. De resultaten van de studie zullen het belang van het volgen van opgelegde interventies vermoedelijk onderbouwen. De uitkomst van dit onderzoek zal steun kunnen geven aan overheidscampagnes bij het verstrekken van informatie aan burgers.

De verwachte **startdatum** is [*13-04-2020*]

De verwachte **einddatum** is [13-08-2020]]

Beantwoord s.v.p. de volgende vragen:

1. Wordt bij het onderzoek gebruik gemaakt van een medisch hulpmiddel?
   ☐ Ja ☐ **Nee** ☐ NVT

   Indien **ja**, dan graag de volgende informatie aanvullen:

   Dit medisch hulpmiddel heeft wel/niet een CE-markering en zal wel/niet worden gebruikt binnen het door de fabrikant beoogde gebruik.

2. Waarom valt volgens u het onderzoek niet onder de Wet Medisch-wetenschappelijk Onderzoek (WMO) met mensen (graag aankruisen wat van toepassing is)?

   ■ Er wordt nader gebruik gemaakt van (medische) gegevens van mensen die reeds vanwege een ander doel zijn of worden vastgelegd.

   ☐ Er wordt nader gebruik gemaakt van lichaamsmateriaal dat reeds vanwege een ander doel (dan wetenschappelijk onderzoek) is of wordt verzameld of dat overblijft na diagnostiek of behandeling.

   ■ Anders, namelijk _____Niet belastend vragenlijst onderzoek_________

3. Betreft het een onderzoek waarbij patiënten / proefpersonen worden geïncludeerd?

   ■ Ja ☐ Nee

   Indien **ja**, geef aan hoeveel deelnemers het betreft en typeer de populatie:

   In deze studie zullen [*X aantal deelnemers worden geïncludeerd / de gegevens van X aantal mensen worden gebruikt*].

De populatie betreft: 170 patiënten met de verdenking COVID-19 (bijna alle presentaties voor de longziekten op de SEH, omdat als iemand hoest als verdacht wordt beoordeeld) worden geïncludeerd en van deze patiënten worden gegevens gebruikt. We verwachten een verdeling van 40% negatieve en 60% positieve Covid-19 patiënten.

4. Betreft het onderzoek waarbij proefpersonen aan (een) handeling(en) worden onderworpen of hen gedragsregels worden opgelegd (inclusief het afnemen van vragenlijst(en))?

   ■ Ja ☐ Nee

Indien **ja**, welke handeling(en) of gedragsregels gebeuren *niet* in het kader van de standaardbehandeling van de proefpersoon?

Indien **ja**, voeg dan de vragenlijsten en/of interviewvragen toe aan uw indiening!

5. Wat is de (verwachte) belasting voor de deelnemers:

■ Belasting in tijd/duur, te weten ___15 minuten ____________

■ Psychologische belasting, te weten _Minimaal, patiënt moet zich tijdens zijn presentatie op de SEH concentreren om de vragen te beantwoorden.

____

☐ Lichamelijke belasting, te weten ___Nee___________

6. Hoe worden de deelnemers benaderd en geïnformeerd?

Beschrijving
Patiënten worden op de SEH geïnformeerd door een arts-assistent over de belasting, aantal vragenlijsten en de inhoud van de vragen. Dit gebeurt in de wachttijd tot het aanvullend onderzoek van de reguliere zorg bekend is.

Bij deze niet-WMO aanvraag is wel/geen informatiebrief en toestemmingsverklaring van toepassing.

Er zal een waiver gebruikt worden op de SEH vanwege meerdere onderzoeken die lopen op de SEH mbt het corona virus.
Wel zullen we de patienten toestemming vragen in de eerste vraag in de vragenlijst voor deelname aan het onderzoek.

Let op: óók bij dossieronderzoek moet informatie worden verstrekt over het onderzoek en moet schriftelijke toestemming worden gevraagd, tenzij dit in de praktijk onmogelijk is.

Zo **nee**, de reden hiervoor is dat

☐ het aantal deelnemers aan wie toestemming moet worden gevraagd zo groot is dat het onmogelijk is om dit uit te voeren.
Let op: u heeft onder de AVG een inspanningsverplichting!

☐ de gegevens / het lichaamsmateriaal op geen enkele manier meer te herleiden zijn/is naar een persoon.

☐ andere reden, namelijk _____________

Gegevens onderzoeker(s)

Gegevens hoofdonderzoeker
Heer/mevrouw, titels en naam: Kuiper- van der Valk, MD, PhD
Functie/afdeling/instelling: AIOS + Post Doc Longziekten
E-mailadres: h.kuiper-vandervalk@franciscus.nl
Postadres (inclusief kamernummer): Kleiweg 500, 3045 PM, Rotterdam. Kamer 00D41

Handtekening:

Gegevens indiener *[als deze verschilt van de hoofdonderzoeker, bijv., uitvoerend onderzoeker, promovendus, aios, contactpersoon]*
Heer/mevrouw, titels en naam:
Functie/afdeling/instelling:
E-mailadres:
Postadres (inclusief kamernummer):
Handtekening:

Wie is de **opdrachtgever** van het onderzoek?

(*Franciscus Gasthuis en Vlietland (ziekenhuis)*

Factuurgegevens
Voor aanvragen afkomstig van een opdrachtgever die *niet* is aangesloten bij MEC-U (zowel andere zorginstellingen als industrie) worden kosten in rekening gebracht.
Het factuuradres is:

Franciscus Gasthuis & Vlietland
JPM Kuiper van der Valk
Kostenplaatnummer: 800521
Kleiweg 500 3045 PM Rotterdam
Als de factuurgegevens niet compleet zijn, dan nemen wij uw aanvraag niet in behandeling!

heeft verwijderd: ¶

Betreft het een promotieonderzoek of een deel van een promotieonderzoek?
☐ Ja ☐ **Nee**

Zo **ja**, naam promovendus / instelling / afdeling:

Betreft het onderzoek een afstudeerproject of een stageonderzoek?
☐ Ja ☐ **Nee**

Zo **ja**, naam student / stagiair / opleidingsinstituut / opleiding:

Eventuele overige betrokkene(n)

Naam	Instelling(en):	Afdeling(en)

A.W.M. Evers, Prof, PhD**

M. Middendorp, Assoc. Prof, PhD**

Dr. J.C.C.M. in ’t Veen, MD, PhD*

*Department of Pulmonary Medicine Franciscus Gasthuis & Vlietland

** Department of Medical and Neuropsychology University Leiden

Overzicht documenten

Bijgevoegd vindt u de documenten behorend bij de niet-WMO aanvraag.
De gevraagde documenten graag **in *aparte* bijlage(n)** aanleveren**!**

Bijlagen	Bijgevoegd	N.v.t.
A1 Begeleidend schrijven (brief of e-mail)*	X	
C1 Onderzoeksprotocol*	X	
E1-2 Informatiebrief incl. toestemmingsverklaring #1		
E3/4 Eventueel (ander) wervings- of voorlichtingsmateriaal		
F1 Vragenlijst(en) #2		
K6 Vragenlijst niet-WMO*	X	
**Overige bijlagen**	**Bijgevoegd**	

* Verplichte documenten
#1 Verplicht (indien van toepassing, zie vraag 6)
#2 Verplicht als u vragenlijsten of interviews afneemt (zie vraag 4)
