# Supplementary material for: Case-control study of patient characteristics, knowledge of the COVID-19 disease, risk behaviour and mental state in patients visiting an emergency room with COVID-19 symptoms in the Netherlands
Source: PLoS One. 2021 Apr 28;16(4):e0249847. doi: 10.1371/journal.pone.0249847 (PMC8081234; doi:10.1371/journal.pone.0249847)
Supplement: S3 Protocol — (DOCX) [file pone.0249847.s005.docx]

***Woord vooraf:***

*Wetenschappelijk onderzoek dat niet onder de Wet Medisch Wetenschappelijk Onderzoek met Mensen (WMO) valt, maar waar wel patiënten en/of patiëntgegevens bij betrokken zijn, moet worden getoetst door de Advies Commissie Wetenschap (ACW) van Franciscus.*

*Deze toetsing richt zich op de vraag of het onderzoek is opgezet en wordt uitgevoerd volgens geldende wet- en regelgeving en richtlijnen, of de belasting voor de deelnemer gerechtvaardigd is, of het onderzoek uitgevoerd kan worden binnen Franciscus en of de wetenschappelijke kwaliteit voldoende is.*

*Om tot een juiste beoordeling te komen dient een niet WMO protocol duidelijk onderstaande informatie te bevatten, waarbij het ook mogelijk is een protocol in te dienen waarin de punten letterlijk terugkomen.*

*Meld je studie aan voor lokale toetsing via Castor Study Management System (SMS) via* [*https://franciscus.castorsms.com/user/login*](http://iprova.sfg.net/management/hyperlinkloader.aspx?hyperlinkid=8d31512b-dc52-400f-b64b-7d308e96e746)*. Kijk voor meer informatie op intranet bij ACW of mail wetenschapsbureau@franciscus.nl*

***Gebruik van dit format:***

*Gebruik dit format als leidraad. Vul bij ieder hoofdstuk iets in, ook als het niet van toepassing is (dan is ‘n.v.t.’ voldoende). De inhoudelijke kwaliteit wordt beoordeeld door de ACW. Indien nodig krijg je feedback ter verbetering / verheldering van je protocol.*

*De schuingedrukte tekst is instructie. Bij indiening van het protocol kan je deze tekst weghalen zodat een logisch geheel wordt.*

*Hulp nodig bij het opstellen van je protocol?
Mail dan naar:* [*wetenschapsbureau@franciscus.nl*](http://iprova.sfg.net/management/hyperlinkloader.aspx?hyperlinkid=e2b7d71d-6c7b-4677-8b02-7c1d9b574268) *of kom even langs – locatie Gasthuis 00A17 of maak een afspraak.
Tel: 010-461 1755*

**Format onderzoeksprotocol Niet WMO-plichtig onderzoek** (augustus 2018)

| Datum / Versie | *3-04-2020* |
| --- | --- |
| **Algemene gegevens** |  |
| Titel onderzoek | ***P****atient’s knowledge* ***A****nd behavior o****N*** *the COVID-19 disease and as* ***DE****ter****MI****nants of* ***C****ontamination* |
| Acroniem/ korte studie titel | ***PANDEMIC****- study* |
| Intern studienummer |  |
| Opdrachtgever / verrichter | *J.P.M. Kuiper-van der Valk, Franciscus Gasthuis en Vlietland* |
| Indiener | *, longarts Franciscus Gasthuis* |
| (Lokale) Hoofdonderzoeker Franciscus | *Titel(s), voorletters en achternaam: J.P.M. Kuiper- van der Valk*  *Functie & afdeling: Longarts io, post-doc , afdeling longziekten* |
| Overige betrokken onderzoekers in Franciscus | *Titel(s), voorletters en achternaam: J.C.C.M in ‘t Veen*  *Functie & afdeling: longarts, longziekten* |
| Betrokken onderzoeker buiten Franciscus (indien van toepassing) | *NVT*  *Prof. A.W.M. Evers, Assoc. Prof M. Middendorp* |
| Het onderzoek wordt uitgevoerd in het kader van: | *Post-Doc* |
|  |  |
| Bijlagen | *<* ***Graag aangeven welke bijlage(n) is/zijn bijgevoegd (indien nvt dan weglaten), inclusief versienummer en/of datum:*** *Voor niet WMO-plichtig onderzoek:*  *A. nWMO-verklaring van METC (alleen als deze reeds is aangevraagd, niet verplicht),  NB: Bij Franciscus geïnitieerd niet-WMO plichtig onderzoek beoordeelt de ACW de WMO plichtigheid en geeft - indien geen twijfel over de niet-WMO status – de niet WMO verklaring af.*  *A. Positief advies CGR/DCTF (indien farmaceutische nWMO studie, zie* [*http://nwmostudies.nl*](http://iprova.sfg.net/management/hyperlinkloader.aspx?hyperlinkid=d4864667-5b28-411e-95fa-b1a1f344264f)*)*  *E1. Patiënteninformatie mét Franciscus logo en lokale gegevens (indien van toepassing)*  *E2. Informed consent mét Franciscus logo (indien van toepassing)*  *E3: Eventuele advertentieteksten of ander wervingsmateriaal (indien van toepassing)*  *F1. Vragenlijsten (indien van toepassing)*  *F2. Patiëntendagboeken (indien van toepassing)*  *I1. Lijst deelnemende centra (indien van toepassing)*  *I3. CV Franciscus-hoofdonderzoeker*  *I4. CV Franciscus-coördinerend onderzoeker*  *K3. Contracten/overeenkomsten (indien geen standaard format gebruikt worden deze door onze Franciscus-jurist beoordeeld, indien wel standaard format gebruikt door ACW lid)>*  *O. Financieel overzicht: stelt Wetenschapsbureau op, indien extra zorg activiteiten of inzet van personeel relevant, dan neemt WB contact op met onderzoeker voor evt. vragen* |

Inhoud

[1. Introductie 4](#_Toc525819873)

[2. Doelstelling/ onderzoeksvraag en studie design 4](#_Toc525819874)

[Onderzoeksdoel(en) 4](#_Toc525819875)

[Studie design 4](#_Toc525819876)

[3. Populatie 4](#_Toc525819877)

[Inclusie criteria 4](#_Toc525819878)

[Exclusie criteria 4](#_Toc525819879)

[Aantal proefpersonen/ sample grootte 4](#_Toc525819880)

[Duur van de studie 5](#_Toc525819881)

[4. Procedures van onderzoek en evt. behandelingen 5](#_Toc525819882)

[Studie procedure 5](#_Toc525819883)

[5. Variabelen en meetmethoden 5](#_Toc525819884)

[Primaire uitkomstmaat (afhankelijke variabele) 5](#_Toc525819885)

[Secundaire uitkomstmaat 5](#_Toc525819886)

[Overige variabelen (onafhankelijke variabelen) 5](#_Toc525819887)

[Overzicht variabelen en meetinstrumenten 6](#_Toc525819888)

[6. Analyses 6](#_Toc525819889)

[Statistische analyses 6](#_Toc525819890)

[7. Ethische aspecten 6](#_Toc525819891)

[Motivatie niet WMO plichtig onderzoek 6](#_Toc525819892)

[Belasting voor de proefpersoon 6](#_Toc525819893)

[Risico voor de proefpersoon 6](#_Toc525819894)

[8. Administratieve aspecten 7](#_Toc525819895)

[Privacy en databeheer 7](#_Toc525819896)

[Publicatiebeleid 9](#_Toc525819897)

[9. Financieel 9](#_Toc525819898)

[Overige opmerkingen 9](#_Toc525819899)

[Referenties 9](#_Toc525819900)

# 1. Introductie

# 2. Doelstelling/ onderzoeksvraag en studie design

## Onderzoeksdoel(en)

##

## Studie design

**Background:** Coronavirus disease 2019 (COVID-19) is the most serious respiratory virus pandemic since the 1918 H1N1 influenza pandemic. In the absence of a COVID-19 vaccine, non-pharmaceutical interventions (NPIs) are crucial to reduce virus transmission. The importance to adhere to the interventions, imposed by the government, is considered crucial. Knowledge on contamination risks and on the severity, the possible consequences, the chance on recovery and long-term consequences of the disease will differ between individuals. Risk behavior will influence adherence to interventions such as case isolation, home quarantine, social distancing (especially of those over 70 years of age), avoiding mass gathering and social distancing.

We hypothesize that patient’s knowledge on the COVID-19 virus infection and (risk) behavior will be related to infection risk to COVID-19 virus.

**Aim**: The primary aim is to study patient’s knowledge of the disease and their risk behavior. The secondary aim is to relate the level of this knowledge and risk behavior (sticking to NPI’s) to contamination.

**Method:** We will include at least 170 patients (2 groups, difference in illness perception of 20%, 90% power and alpha 0.05) consecutive patients subject to COVID-19 infection screening at the emergency room. After giving informed consent, patients will be asked to fill out validated questionnaires (on an iPad with disposable cover) on knowledge of the disease (*Illness Perception Questionnaire, Leventhal*), on patients health (*The Short Form (36) Health Survey*) and on risk behavior. The relation between disease insight and risk behavior will be investigated in the COVID-19 positive group and will be compared to both, the COVID-19 negative group and to historical data. The study is not burdensome to the patient (not subject to the WMO) and is feasible on the short term.

**Results:** The results of the questionnaires will be presented descriptively and will be correlated to contamination risks. The results of the study will putatively substantiate the importance of adhering to imposed interventions. The outcome of this study will be able to support government campaigns in providing information to citizens.

## Nederlandse samenvatting

Achtergrond: Het Coronavirus 2019 (COVID-19) is de meest ernstigste respiratoire pandemie sinds de H1N1-grieppandemie van 1918. Bij afwezigheid van een COVID-19-vaccin zijn niet-farmaceutische interventies (NFI's) cruciaal om de overdracht van virussen te verminderen. Het belang van het naleven van de door de overheid opgelegde interventies wordt van cruciaal belang geacht. Kennis over besmettingsrisico's en over de ernst, de mogelijke gevolgen, de kans op herstel en de langetermijngevolgen van de ziekte zal per persoon verschillen. Risicogedrag zal van invloed zijn op het volgen van interventies zoals het isoleren van personen, quarantaine thuis, sociale afstand houden (vooral van mensen ouder dan 70 jaar) en het vermijden van massa bijeenkomsten.

We veronderstellen dat de kennis van de patiënt over de COVID-19-virusinfectie en (risico) gedrag verband houdt met het infectierisico voor het COVID-19-virus.

Doel: Het primaire doel is om de kennis van de patiënt over de ziekte en hun risicogedrag te bestuderen. Het secundaire doel is om het niveau van deze kennis en risicogedrag (vasthouden aan NFI's) te relateren aan besmetting.

Methode: We zullen ten minste 170 patiënten (2 groepen, verschil in ziekteperceptie van 20%, 90% power en alfa 0,05) includeren. De patiënten worden onderworpen aan een COVID-19-infectie screening op de eerstehulpafdeling of de COVID-19-afdeling. Na het geven van geïnformeerde toestemming, zullen patiënten worden gevraagd om een drietal vragenlijsten in te vullen (op een iPad met wegwerphoes) over kennis van de ziekte, over hun risicogedrag en gemoedstoestand. De relatie tussen ziekte-inzicht en risicogedrag zal worden onderzocht in de COVID-19 positieve groep en zal worden vergeleken met zowel de COVID-19 negatieve groep als met historische gegevens. Het onderzoek is niet belastend voor de patiënt en op korte termijn haalbaar.

Resultaten: De resultaten van de vragenlijsten worden beschrijvend gepresenteerd en worden gecorreleerd aan besmettingsrisico's. De resultaten van de studie zullen het belang van het volgen van opgelegde interventies vermoedelijk onderbouwen. De uitkomst van dit onderzoek zal steun kunnen geven aan overheidscampagnes bij het verstrekken van informatie aan burgers.

# 3. Populatie

##

## Inclusie criteria

Patients ≤ 19 year and subject to COVID-19 infection screening at the emergency room

## Exclusie criteria

## None

## Aantal proefpersonen/ sample grootte

We will include at least 170 patients (2 groups, difference in illness perception of 20%, 90% power and alpha 0.05) consecutive patients subject to COVID-19 infection screening at the emergency room

## Duur van de studie

4 months

# 4. Procedures van onderzoek en evt. behandelingen

## Studie procedure

After giving informed consent, patients will be asked to fill out 3questionnaires (on an iPad with disposable cover) on knowledge of the disease, on patients health/mood and on risk behavior. The relation between disease insight and risk behavior will be investigated in the COVID-19 positive group and will be compared to both, the COVID-19 negative group and to historical data.

# 5. Variabelen en meetmethoden

## Primaire uitkomstmaat (afhankelijke variabele)

- Covid infection

## Secundaire uitkomstmaat

- Illness perception
- Risk behavior
- Mood

## Overige variabelen (onafhankelijke variabelen)

- Age

- Gender

- Education

- Family composition

- Profession

# 6. Analyses

## Statistische analyses

All data will be presented quantitatively. A p< 0.05 will be regarded as statistically significant. All analysis will be performed using SPSS v.25 or later. Variables will be measured on a nominal or ordinal scale and will be summarized as proportions (or odds ratio or relative risk, as appropriate) with 95% confidence intervals.

# 7. Ethische aspecten

## Motivatie niet WMO plichtig onderzoek

## This study is not subject to WMO , because it is a questionnaire study without burdensome questions for the patients.

## Belasting voor de proefpersoon

## This study is not burdensome for the patients. The patients will be asked to fill out 3 short questionnaires on a iPad in the waiting time during their emergency room visit.

## Risico voor de proefpersoon

There are no risks for the patients.

# 8. Administratieve aspecten

| Privacy en databeheer | |
| --- | --- |
| **8.1**  Wordt in het kader van dit onderzoek aan patiënten/betrokkenen toestemming gevraagd voor het gebruik van hun (medische) gegevens? | There will be a general PIF for all COVID-19 research |
| **8.2**  Indien geen toestemming wordt gevraagd: licht toe waarom geen toestemming wordt gevraagd | Not applicable |
| **8.3**  Is er sprake (geweest) van een behandelrelatie tussen de onderzoeker(s) of de afdeling van de onderzoeker(s) en de patiënten van wie de status wordt ingezien? (zie ook hierna onder 8.6 t/m 8.8) | No |
| **8.4**  Van hoeveel patiënten worden gegevens gebruikt? | 170 |
| **8.5**  Wordt er van het gebruik van (medische) gegevens voor wetenschappelijke doeleinden aantekening gemaakt in de status van de desbetreffende patiënt(en)? | We will make a note in the EPD that these pastients participate in the PANDEMIC-study |
| **8.6**  Door wie (naam, functie) worden de benodigde gegevens uit de patiëntendossiers gehaald? | JPM Kuiper-van der Valk, PhD, MD  Post-Doc position |
| **8.7**  Zijn de personen die de benodigde gegevens uit de patiëntendossiers halen hiertoe gerechtigd uit hoofde van een behandelrelatie met de desbetreffende patiënt (of is hiervoor toestemming aan de patiënt gevraagd)? | Yes |
| **8.8**  Indien het antwoord op vraag 8.7 ‘nee’ is: Staan de personen die de gegevens uit de patiëntendossiers halen onder directe supervisie van een behandelaar die uit hoofde van de behandelovereenkomst wel gerechtigd is tot inzage in de dossiers? | Not applicable |
| **8.9**  Worden er tot de persoon herleidbare gegevens ter beschikking gesteld aan de onderzoeker(s)? | No |
| **8.10**  Indien codering plaatsvindt: wanneer vindt codering plaats, door wie en op welke wijze (hoe is deze codering opgebouwd)? | In Castor with a coded list by J.P.M. Kuiper- van der Valk |
| **8.11**  Indien van toepassing: waar worden gecodeerde gegevens opgeslagen (in welk systeem? en is dit systeem getoetst volgens een PIA (Privacy Impact Analyse)? | Castor EDC |
| **8.12**  Indien van toepassing: waar wordt de sleutel, waarmee gecodeerde gegevens zijn te herleiden tot de patiënt, bewaard (sharepoint / afdelingsschijf)? | Yes, on an encrypted disk |
| **8.13**  Indien van toepassing: door wie wordt de sleutel van de gecodeerde data beheerd/ wie heeft toegang tot de sleutel ent tot de brondocumenten en eventuele andere tot de persoon herleidbare gegevens? | J.P.M. Kuiper-van der Valk |
| **8.14**  Welke technische en organisatorische maatregelen zijn er getroffen ter voorkoming van verlies, diefstal of ongeautoriseerd gebruik van de onderzoeksdata? | Data is encrypted on the computer, Franciscus Gasthuis |
| **8.15**  Vindt er uitwisseling (zowel uitgifte als opvragen extern) van (onderzoeks)gegevens plaats met (een) andere instelling(en) binnen Nederland en/of de EU? En zo ja, op welke wijze? | No |
| **8.16**  Vindt er uitwisseling van (onderzoeks)gegevens plaats met (een) andere instelling(en) buiten de EU? | No. |
| **8.17**  Wordt aan de desbetreffende patiënten toestemming gevraagd voor het uitwisselen van persoonsgegevens met een land/ landen buiten de EU? | No. |
| 8.18  Hoe lang worden de gegevens van het onderzoek bewaard? | 15 years |

## Publicatiebeleid

# The final results of the study will be published in article form.

# 9. Financieel

Is er een (financiële) vergoeding beschikbaar voor dit onderzoek?: *No*

#

# Referenties

1. James Holland Jones and  [Marcel Salathé](https://www.ncbi.nlm.nih.gov/pubmed/?term=Salath%26%23x000e9%3B%20M%5BAuthor%5D&cauthor=true&cauthor_uid=19997505). Early Assessment of Anxiety and Behavioral Response to Novel Swine-Origin Influenza A(H1N1). PLoS One2009; 4(12): e8032.
2. Geldsetzer P. Use of Rapid Online Surveys to Assess People's Perceptions During Infectious Disease Outbreaks: A Cross-sectional Survey on COVID-19. J Med Internet Res. 2020 Apr 2;22(4):e18790.
