# Supplementary material for: Case-control study of patient characteristics, knowledge of the COVID-19 disease, risk behaviour and mental state in patients visiting an emergency room with COVID-19 symptoms in the Netherlands
Source: PLoS One. 2021 Apr 28;16(4):e0249847. doi: 10.1371/journal.pone.0249847 (PMC8081234; doi:10.1371/journal.pone.0249847)
Supplement: S1 File — (PDF) [file pone.0249847.s008.pdf]

Quantitative variables	11	Explain how quantitative variables were handled in the analyses. If applicable, describe which groupings were chosen and why	V 5
Statistical methods	12	*(a)* Describe all statistical methods, including those used to control for confounding	V 5
		(b) Describe any methods used to examine subgroups and interactions	V 5
		(c) Explain how missing data were addressed	V 4
		(d) Cohort study—If applicable, explain how loss to follow-up was addressed<br>*Case-control study*—If applicable, explain how matching of cases and controls was addressed<br>*Cross-sectional study*—If applicable, describe analytical methods taking account of sampling strategy	—
		(e) Describe any sensitivity analyses	V 5
**Results**			
Participants	13*	(a) Report numbers of individuals at each stage of study—eg numbers potentially eligible, examined for eligibility, confirmed eligible, included in the study, completing follow-up, and analysed	V 4
		(b) Give reasons for non-participation at each stage	V 4
		(c) Consider use of a flow diagram	V 5
Descriptive data	14*	(a) Give characteristics of study participants (eg demographic, clinical, social) and information on exposures and potential confounders	V 6
		(b) Indicate number of participants with missing data for each variable of interest	V 5
		(c) *Cohort study*—Summarise follow-up time (eg, average and total amount)	—
Outcome data	15*	*Cohort study*—Report numbers of outcome events or summary measures over time	V 5
		Case-control study—Report numbers in each exposure category, or summary measures of exposure	V 6-10
		Cross-sectional study—Report numbers of outcome events or summary measures	
Main results	16	*(a)* Give unadjusted estimates and, if applicable, confounder-adjusted estimates and their precision (eg, 95% confidence interval). Make clear which confounders were adjusted for and why they were included	V 6-10
		(b) Report category boundaries when continuous variables were categorized	V 6-10
		(c) If relevant, consider translating estimates of relative risk into absolute risk for a meaningful time period	—

Continued on next page
