# Supplementary figures and images for: Case-control study of patient characteristics, knowledge of the COVID-19 disease, risk behaviour and mental state in patients visiting an emergency room with COVID-19 symptoms in the Netherlands
Source: PLoS One. 2021 Apr 28;16(4):e0249847. doi: 10.1371/journal.pone.0249847 (PMC8081234; doi:10.1371/journal.pone.0249847)

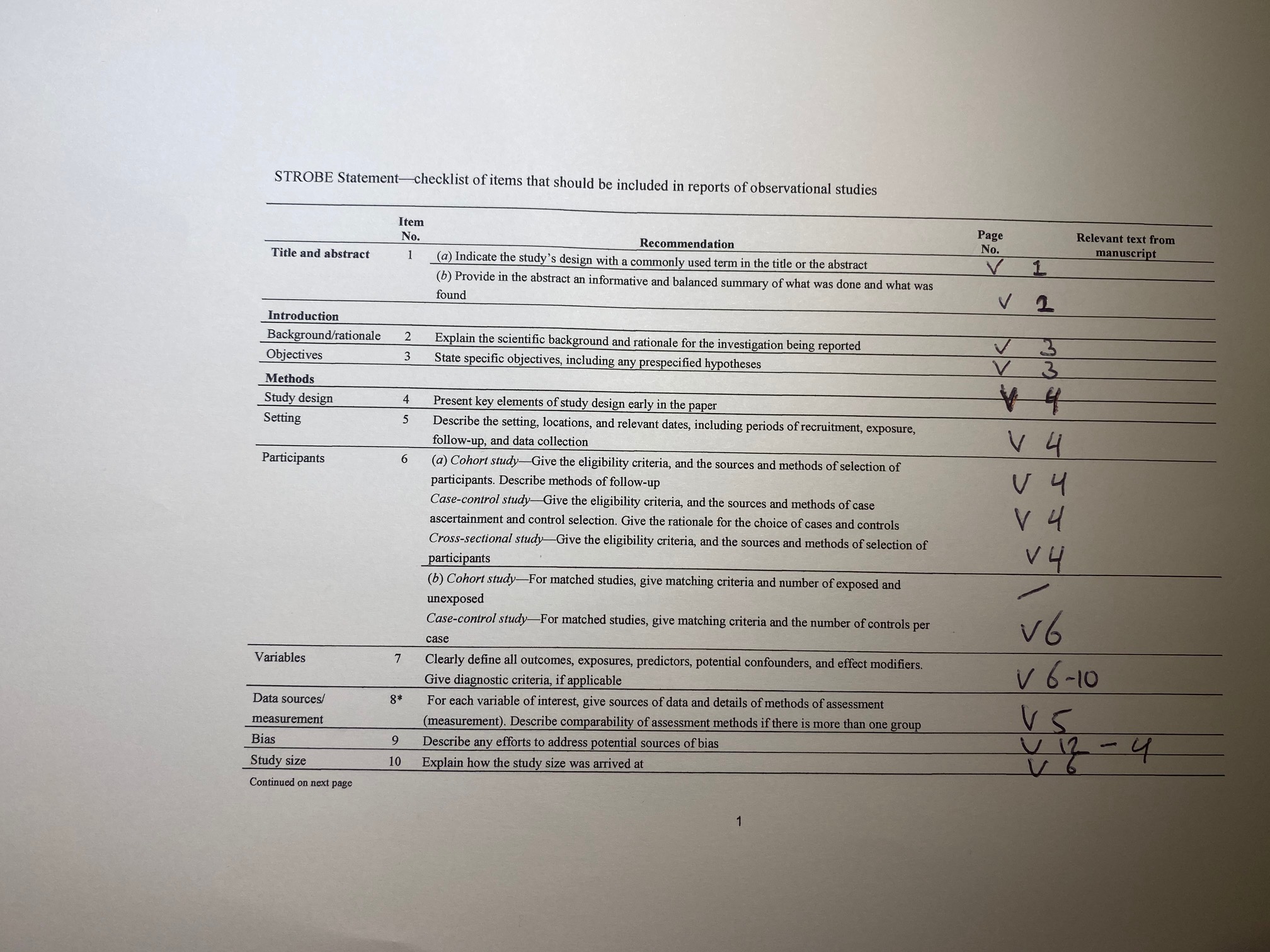

Supplement: S2 File — (JPG) [file pone.0249847.s009.jpg]

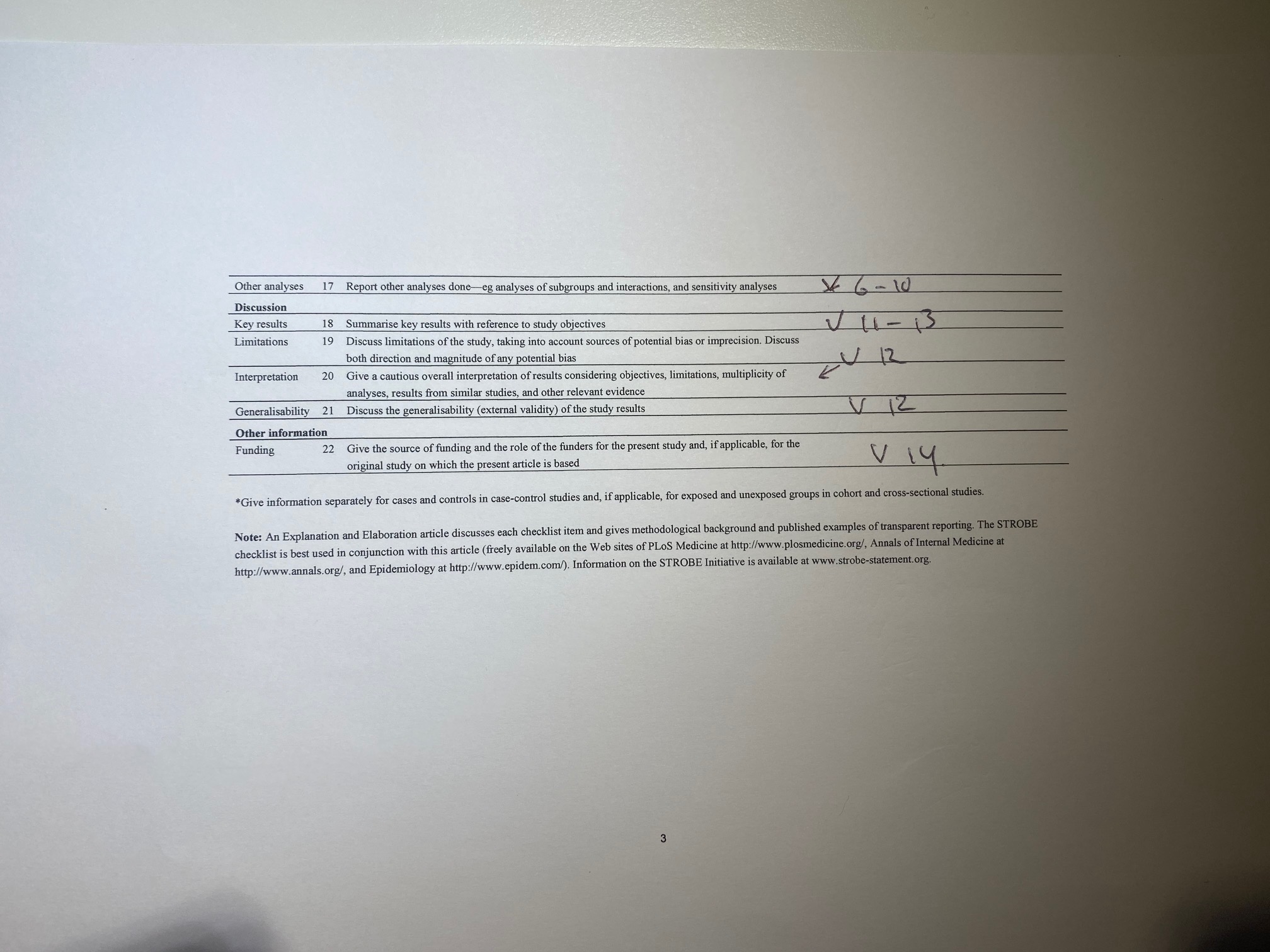

Supplement: S3 File — (JPG) [file pone.0249847.s010.jpg]
