# Supplementary material for: Case-control study of patient characteristics, knowledge of the COVID-19 disease, risk behaviour and mental state in patients visiting an emergency room with COVID-19 symptoms in the Netherlands
Source: PLoS One. 2021 Apr 28;16(4):e0249847. doi: 10.1371/journal.pone.0249847 (PMC8081234; doi:10.1371/journal.pone.0249847)
Supplement: Attachment — Submitted filename: renamed_dec77.docx [file pone.0249847.s011.docx]

**COMMENTS – Manuscript PONE-D-20-28005**

Title: “Case- control study of patient characteristics, knowledge of the COVID-19 disease, risk behaviour and mental state in patients visiting an emergency room with COVID-19 symptoms in the Netherlands”

**Important note: This review pertains only to ‘statistical aspects’ of the study and so** **‘****clinical aspects’ [like medical importance,** **relevance of the study,** **‘clinical significance and** **implication(s)’ of the whole study, etc.] are to be evaluated [should be assessed] separately/independently. Further please note that any ‘statistical review’ is generally done under the assumption that (such) study specific methodological [as well as execution] issues are perfectly taken care of by the investigator(s). This review is not an exception to that and so does not cover clinical aspects {however, seldom comments are made only if those issues are intimately / scientifically related & intermingle with ‘statistical aspects’ of the study}. Agreed that ‘statistical methods’ are used as just tools here, however, they are vital part of methodology [and so should be given due importance].**

**COMMENTS:** It is (being KAP type cross-sectional survey only) a fairly simple [and so straight forward] study as ‘The primary aim of this study was to investigate “Patient characteristics, knowledge of the COVID-19 disease, risk behaviour and illness perception in patients visiting an emergency department in the Netherlands during the COVID-19 pandemic”. However, as said in (lines 63-64) ‘Abstract-Conclusion’ that “This is the first (large) study that investigates these’ is not true. But in any case, I request the authors to consider/note following points:

It may please be noted {kindly confirm from field experts} that patients having SARS-Cov-2 antibodies are not capable to spread the disease [unless IgM result shows status of infection]. If that is so, then how correct is to combine them? with ‘Patients with a positive nasal Polymerase chain reaction (PCR) swab to SARS-Cov-2’ which are real ‘COVID-19 positive’.

Though measures/tools used as “Indicators/Measures of knowledge, risk behaviour, and illness perception of COVID-19” (lines 155-56), are appropriate, most of them yield data that are in [most likely] ‘ordinal’ level of measurement [and not in ratio level of measurement for sure {as the score two times higher does not indicate presence of that parameter/phenomenon as double}]. Then application of suitable non-parametric test(s) is/are indicated/advisable [even if distribution may be ‘Gaussian’ (i.e. normal) in these (such) cases. Therefore, as said in lines 157-8 that ‘differences in terms of these factors were compared between 3 groups (those tested positive for SARS-Cov-2, those tested negative, and the control group) using the One-Way ANOVA test’ is not correct and it is indicated/advisable to use non-parametric ‘One-Way ANOVA’ namely Kruskal-Wallis test.

Please read the following [from famous text book]:

“Inferential statistics (i.e. hypothesis testing + estimation of CI) is built on the population model (i.e. the underlying assumption is that there is a population and we are dealing with random sample(s) drawn from that population). Although in clinical trial (involving at least two groups) we do not really deal with random samples, ‘allocation’ to treatment groups is ‘randomly’ done which enable us to evoke the population model and we can use inferential statistics safely. But when there is only one group or in studies even with two/more groups ‘random allocation’ is out-of- question [like internal grouping as in this case] and with ‘non-random’ selection, it may be questionable to use inferential statistics [even if you have two measurement sets as ‘pre-post’].

By this I do not advice “not to use inferential statistics here”, but just to keep this limitation in mind while interpreting results as there is no guarantee of representation of population {example, line 336: The lower average age in this study compared to the SARS-Cov-2-infected populations reported in other studies}.

Test used to analyse data displayed in Table 2 [Group comparison for non-pharmaceutical interventions (NPIs) and SARS-Cov-2 infection in the positive-tested, negative-tested, and control groups] is not mentioned anywhere and so the question is ‘how ANOVA is applied as most data are categorical’ but if Chi-square is used then ‘how zero frequency (rather all low frequencies are/) is dealt with’ [remember that this a scientific/academic document and so all details should be clearly communicated].

Implications of this study [in backdrop of ‘Added value of this study’ described in lines 396-40] are questionable, in my opinion {though it is true (line 71-72) that ‘Knowledge about the coping of the population during the COVID-19 pandemic is very important, certainly also in the perspective of a possible second outbreak of COVID-19’}. Few things/findings are ‘very obvious’ [ex. Line 186-7: ‘*Significantly more patients infected with SARS-Cov-2 work in a vital profession compared to uninfected patients and the control group (p-value 0.04), and most of them work in the medical sector*’].
