# Supplementary material for: Case-control study of patient characteristics, knowledge of the COVID-19 disease, risk behaviour and mental state in patients visiting an emergency room with COVID-19 symptoms in the Netherlands
Source: PLoS One. 2021 Apr 28;16(4):e0249847. doi: 10.1371/journal.pone.0249847 (PMC8081234; doi:10.1371/journal.pone.0249847)
Supplement: Attachment — Submitted filename: Letter_reviewer2.docx [file pone.0249847.s013.docx]

Dear Editor

We would like to respond to the comment of the referee.

1. *"While answering my comment, it is said that “Fortunately, the new results of this statistical method did not change our conclusions” is very good but remember that it does not indicate that any will do. Methodology used should be appropriate [always use which is indicated / most desired."*

I fully agree with you. I am very pleased that you pointed this out to me, and indeed the correct methodology is very important. We want to apologize for the word choice we made.

Looking forward to hear from you.

Kind regards,

Hanna van der Valk
