# Supplementary material for: Case-control study of patient characteristics, knowledge of the COVID-19 disease, risk behaviour and mental state in patients visiting an emergency room with COVID-19 symptoms in the Netherlands
Source: PLoS One. 2021 Apr 28;16(4):e0249847. doi: 10.1371/journal.pone.0249847 (PMC8081234; doi:10.1371/journal.pone.0249847)
Supplement: Attachment — Submitted filename: Letter_reviewer3.docx [file pone.0249847.s014.docx]

Dear Editor

We would like to respond to the comment of the referee.

*Reviewer #1: COMMENTS: As already said, it being a KAP type cross-sectional survey only [is fairly simple and so straight forward], there is hardly anything to comment, mention/point-out critical observation(s) or suggest few things for further improvement.
However, I just felt that the fact ‘this study also includes comparison between three groups [Negative-tested, Positive-tested, Control] is not adequately mentioned [except line 64 of ‘Abstract-Conclusion’ that “This is one of the first (large) study that investigates and compares patient characteristics”]*

We agree with you. As you suggest, we mentioned now more adequately in our manuscript the fact that this study describes the variables in patients visiting an emergency department in the Netherlands during the COVID-19 pandemic and that we also made a comparison between the “COVID-19 suspected” (positive and negative tested group) with the “COVID-19 not suspected” (control group) and in the “COVID-19 suspected” group, the positive and negative tested patients.

We added the following sentences to the manuscript:

*Abstract (line 46-49)*

*The primary aim of this study was to investigate these variables in patients visiting an emergency department in the Netherlands during the COVID-19 pandemic and to compare the “COVID-19 suspected” (positive and negative tested group) with the “COVID-19 not suspected” (control group) and to compare in the “COVID-19 suspected” group, the positive and negative tested patients.*

Material and methods (line 160-162)

*A comparison is made between the “COVID-19 suspected” (positive and negative tested group) with the “COVID-19 not suspected” (control group) and in the “COVID-19 suspected” group, the positive and negative tested patients.*

We clarified this also in the discussion (line 314-317)

*The “COVID-19 suspected” (positive and negative tested group) was compared with the “COVID-19 not suspected” (control group) and in the “COVID-19 suspected” group, the positive and negative tested patients were compared.*

We really hope -by addressing you comment- that we improved the message of the manuscript. We are looking forward to hear from you.

Kind regards,

Hanna van der Valk
